# Eosinophils and Oral Squamous Cell Carcinoma: A Short Review

**DOI:** 10.1155/2009/310132

**Published:** 2009-12-16

**Authors:** C. P. Martinelli-Kläy, B. R. R. N. Mendis, T. Lombardi

**Affiliations:** Laboratory of Oral and Maxillofacial Pathology, Division of Stomatology and Oral Surgery, Faculty of Medicine, University of Geneva, 1205 Geneva, Switzerland

## Abstract

The eosinophil cell has been related as a prognostic indicator for cancers. However, its exact function in tumour behaviour is still not clearly defined. In the oral cavity the presence of eosinophils can be a favourable prognostic indicator as well as it may be associated with a poor prognosis. In this short review, we briefly summarize the role of the eosinophils in the general context of immunoregulation and its relation to oral squamous cell carcinoma.

## 1. Introduction

The vast majority of cancers in the oral cavity are squamous cell carcinomas (SCC)—its evolution is influenced by host immune response cells (e.g., CD8^+^ T, CD4^+^ T, natural killer cells-NK, dendritic cells-DC, macrophages, and eosinophils) [1]. 

**Figure 1:**
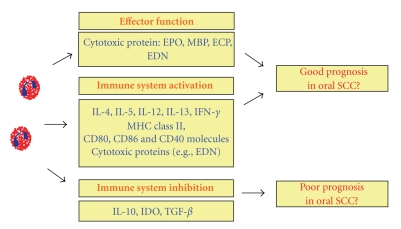
Eosinophil function in the immune response. The eosinophils can stimulate or inhibit the immune response, leading to a probable good or poor prognosis, respectively. EPO: Eosinophil peroxidase; MBP: Major basic protein; ECP: Eosinphil cationic protein; EDN: Eosinophil-derived neurotoxin; IL (Interleukin)-4, IL5, IL-10, IL-12, IL-13; IFN-*γ*: Interferon-*γ*; MHC II: histocompatibility complex II molecules; IDO: indoleamine 2, 3-dioxygenase; TGF-*β*: Transforming growth factor-*β*.

The eosinophils are considered as destructive effector leukocytes with cytotoxic activities mainly implicated in parasitic infections (e.g., helminthic infections) and allergic diseases (e.g., bronchial asthma, allergic dermatitis, etc.). However, studies have shown that they can also be involved in tissue remodelling and in innate and acquired immunity response modulation [2, 3] (Figure 1). 

Under diverse stimuli (e.g., infections, tumours, etc.), the eosinophils are able to release different substances, such as, eosinophil cationic protein (ECP), major basic protein (MBP), eosinophil peroxidise (EPO), eosinophil-derived neurotoxin (EDN), IL-1, IL-2, IL-4, IL-5, IL-6, IL-8, IL-10, IL-12, IL-13, IL-18, interferon (INF)-*γ*, tumor necrosis factor (TNF)-*α*, transforming growth factor (TGF)-*α*, TGF-*β*, chemokines (RANTES, endotaxin-1), platelet-activating factor (PAF)*,* leukotriene C4 (LTC4), neuromediators, and indoleamine 2,3-dioxygenase (IDO) [4, 5]. These substances may cause cell death and induction of inflammatory symptoms as well as contribute to tumour progression or regulation. Furthermore, the eosinophils present membrane receptors (e.g., IL-1, 3, 4, 5, 8, 10, 12, 13, granulocyte monocyte colony-stimulating factor—GM-CSF, IFN-*γ*, TNF-*α*, and macrophage inflammatory protein-1*α*) that confer a survival and recruitment capacity of eosinophils themselves [2]. 

In oral SCC, the exact function of eosinophils still remains unclear. Several studies have shown that eosinophils can be associated with an improved prognosis, but there are other studies however, showing their association with a poor prognosis as well [6, 7].

In this short review, we summarize briefly the role of the eosinophils in the general context of immunoregulation and its relation with oral squamous cell carcinoma.

## 2. The Eosinophil and Its Diverse Functions in the Context of Immunoregulation

Under a specific stimulus, the naïve CD4^+^ T and CD8^+^ T cell can be differentiated, respectively, into Th2 and Tc2 cells and secrete mainly cytokines involved with humoral immunity such as IL-4, IL-5, IL-10, and IL-13 [8, 9]. The initial recruitment and activation of eosinophils towards the tumour microenvironment is principally related with Th2 response, although necrotic cells can stimulate both the migration and the activation of eosinophils [10, 11]. According to the literature, Th2 response may eliminate the cancer on the dependence of eosinophils and macrophages [8, 12–14]. IL-4 and IL-13 are potent inducers of eotaxin chemokines that can explain the eosinophilia associated with Th2 responses [15]. In oral squamous cell carcinoma, the eotaxin expressed by tumour cells and eosinophils were involved in the mechanisms of eosinophil chemotaxis to the tumour [16]. IL-4 presents antiangiogenic properties which could inhibit the tumour growth [17]. Furthermore, IL-5 transgenic mice presented attenuated growth of fibrosarcoma induced by methylcholanthrene [18]. However, IL-4 can have a cell antiapoptotic property which could help tumour growth [19]. IL-13 has also been involved with the antitumour immune response mediated mainly by neutrophils (Gr-1^+^) and macrophages (MAC-3^+^) [20]. However, IL-13 can also inhibit the IFN-*γ* secretion and CD8^+^ cytotoxic T lymphocyte (CTL) activity and compromise the anti-tumour immunity response [21]. 

Studies have also shown that eosinophils can process and present histocompatibility complex II (MHC-class II) molecules [22] and polarize the Th2 response [23]. Upon stimulation with IFN-*γ*, IL-3, and GM-CSF, human eosinophilc cell line differentiated with dibutyryl cyclic AMP (dEoL-1) and human peripheral blood (PB) eosinophils were able to respond to the lymphoid chemokines (e.g., CCL11, CCL21, and CCL25). In addition, cytokine-stimulated dEoL-1 cells expressed human leukocyte antigen (HLA)-DR and costimulatory molecules such as CD80, CD86 and CD40 [24]. Therefore, eosinophils can migrate to the lymphoid chemokine microenvironment and express antigen-presenting cells (APCs)- related costimulatory molecules. However, some experimental models have suggested that eosinophils are inefficient antigen-presenting cells when compared to macrophages [25] or dendritic cells [26]. 

The eosinophil-derived neurotoxin stored in eosinophils granules is able to induce DC maturation and activation through up-regulation of mitogen-activated protein (MAP) kinases, nuclear factor-Kappa B (NF Kappa B) and CD83, CD86 costimulatory molecules, and MHC class II expression [27, 28]. EDN-treated human DCs stimulated Th2 immune response via Toll-like receptor (TLR)2-MyD88 signal [28]. 

Eosinophils can also be related with cytokines of Th1 response [29]. They may express IFN-*γ* as well as IL-4, IL-5 and IL-10 suggesting a subpopulation of human eosinophils that expresses Th1 or Th2 cytokines, respectively [30]. It has been shown that not only the Th1 response is able to eradicate the tumour through the cellular immunity response but also Th2 response via tumour necrosis [12]. Furthermore, IL-12 expressing eosinophils are able to activate Th1 [31]. 

Contrarily, eosinophils are capable to downregulate the antitumour immunity, through mainly IL-10 [32] and IDO production [33]. IL10 is a potent inhibitor of MHC complex and CD80 and CD86 expression on DC as well as it suppresses the DC differentiation. In addition, IL-10 prevents the Th1 and Th2 cytokines production. Contrarily, IL-10 can play a role in the B cell activation and survival [32]. 

Through indoleamine 2,3-dioxygenase production, the eosinophils may also play a role in escape immune surveillance. This enzyme catalyzes the amino acid tryptophan to kynurenine which is able to cause cycle arrest and apoptosis from uncommitted CD4^+^ T cells [33] as well as maintenance of Th2 response [34]. The IDO was correlated with the poor prognosis in the non-small cell lung cancer [35]. 

It has been shown that the eosinophils are capable of producing various substances (e.g., vascular endothelial growth factor-VEGF, fibroblast growth factor*-*FGF, TNF-*α*, GM-CSF, nerve growth factor-NGF, TGF-*β*, and IL-8) that can promote angiogenesis and produce collagenous fibers [36]. In addition, TGF-*β* is considerate as a strong immunosuppressive cytokine—it is capable of inhibiting T cell proliferation and differentiation. TGF-*β* can also inhibit the cytolytic activity of NK [37].

## 3. Prognostic Value of Eosinophils in Oral Squamous Cell Carcinoma

In head and neck SCC, it has been shown that the Th1 response is mainly associated with a better prognosis than those with the Th2 response [38, 39]. According to Argarwal and colleagues [40], early stage of oral SCC expressed mainly INF-*γ* and IL-2 genes (Th1 responses), whereas the advanced stage tumours presented IL-4 and IL-10 expression (Th2 response). In addition, advancing lesions of the tongue squamous cell carcinoma, induced by 4-nitroquinoline-1-oxide, have downregulated Th1-type and upregulated Th2-type cytokine production [41]. 

Regarding studies with eosinophils in the blood, the presence of eosinophils and Th2 cells can be related with tumour progression [42] and poor prognosis [43, 44]. Peripheral blood analyses of intraoral squamous cell carcinoma with a history of tobacco use presented enhanced expression of Th2 cytokine [43]. The metastatic squamous cell carcinoma showed increased blood eosinophilia and elevated serum interleukin-5 levels [44]. However, patients with malignant disease (e.g., acute lymphoblastic leukemia, acute myelogenous leukemia) that underwent stem cell transplantation [45] or patients with cervical cancer treated with whole-pelvic irradiation [46] presented increased blood eosinophilia and better overall survival.

**Figure 2:**
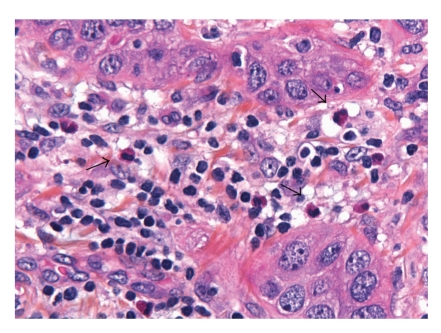
Oral squamous cell carcinoma showing rather numerous eosinophils (black arrows), together with lymphocytes, mingling around nests of nonkeratinized neoplastic oral epithelial cells. Formalin-fixed, paraffin embedded tissue, H&E stained, original magnification ×400.

Tumour-associated tissue eosinophilia (TATE) has been reported in diverse sites [14, 35, 47–53] including the head and neck region [6, 7, 54–65] (Figure 2). 

In the head and neck region, TATE presents controversial results. Some studies have shown that the TATE has a favourable prognosis, suggesting that eosinophils may play a protective role against epithelial tumours [6, 55, 60, 62]. Other studies however, suggest that eosinophils may play a role in promoting epithelial tumour growth accounting for a poor prognosis [7, 57, 58] or even no effect on tumour evolution [54, 56, 59, 63].

With regard to good prognosis, it has been shown that oral SCC patients with TATE presented higher overall survival [6] and less incidence of distant metastasis in head and neck tumours [62]. Nevertheless, Tadbir and colleagues [54] have shown that TATE was not associated with vascular, perineural, muscle invasion, and locoregional metastasis in SCC of the oral cavity. Similar results were observed in nasopharyngeal carcinoma [56] and oral SSC [65] where the eosinophils were not associated with local recurrence, distant metastases, or survival. In another study of laryngeal squamous cell carcinoma, TATE was associated only with the age-TATE-positive patients presented between 50 and 60 years of age, whereas the TATE-negative patients were between 60 and 70 years of age [63]. According to Oliveira and colleagues [65] and Cormier and colleagues [11], the presence of eosinophis in damaged striated muscular fibers of oral cancer or in capsule of B16-F10 melanoma cell-derived tumours, respectively, could be related with tissue remodelling. 

On the other hand, the presence of eosinophils has also been related with a poor prognosis. In oral SCC, eosinophilic infiltration and HLA-DR expression in tumour cells were related to unfavourable prognosis [7]. In the experimental studies of oral carcinoma, the depletion of TATE with anti-IL-5 mAb was associated with a delayed development of the tumours [64]. According to Falconieri and colleagues [61] and Oliveira and colleagues [65], the eosinophil presence in the SCC of the oral cavity was associated with stromal invasion and metastasis [61]. Similar results have been observed in head and neck carcinoma [57, 58] and cervix [51] where eosinophils infiltrate has increased the suspicion of invasion. The presence of >3 eosinophils/high-power field (hpf), ≥5/hpf, and ≥10/10 hpf in cervical incisional biopy and excisional specimens were associated with invasion [51]. 

According to Dorta and colleagues [6], the prognosis related with TATE is controversial due to the different methodology utilized to count tumour-associated tissue eosinophila, thereby prejudicing a comparison of the results. For this reason Alkhabuli and High [66] suggested the counting of eosinophils through a density method, which utilizes the highest density of eosinophils per surface area, in preference to the classic method that counts eosinophils/hpf.

## 4. Conclusion

In general, the presence as well as the state of activation of immunologic cells plays an important role in tumour cell progression. Diverse studies examining eosinophils in tumour development have been carried out. However, the prognostic value of eosinophils in oral carcinoma still remains unclear. Further studies on eosinophils and their state of activation are necessary in order to elucidate these findings.
